# Conceptual Design and Simulation Study of an ROI-Focused Panel-PET Scanner

**DOI:** 10.1371/journal.pone.0072109

**Published:** 2013-08-20

**Authors:** Qingguo Xie, Lu Wan, Xiaoqing Cao, Peng Xiao

**Affiliations:** 1 Biomedical Engineering Department, Huazhong University of Science and Technology, Wuhan, Hubei, China; 2 Wuhan National Laboratory for Optoelectronics, Wuhan, Hubei, China; Virginia Tech, United States of America

## Abstract

Positron emission tomography (PET) is an important imaging modality for clincial use. Conventionally, the PET scanner is generally built to provide a roomy enough transverse field-of-view (FOV) for imaging most adults’ torsos. However, in many cases, the region-of-interest (ROI) for imaging is usually a small area inside the human body. Therefore, to fulfill a PET system which provides an FOV comparable in size to the target ROI seems appealing and more cost effective. Meanwhile, such a PET system has the potential for portable or bedside application with the reduced system size. In this work, we have investigated the feasibility of using dual-headed panel-detectors to build an ROI-focused PET scanner. A novel windowed list-mode ordered subset expectation maximization method was developed to perform the ROI image reconstruction. With this method, the ROI of the object can be reconstructed from the coincidences whose position determined by time-of-flight (TOF) measurements was inside the ROI. Monte Carlo simulation demonstrates the feasibility of detecting lesions not less than 1 cm in diameter, with a 300 ps full width at half maximum timing resolution. As a critical system performance, the impact of TOF information on image quality has been studied and the required TOF capability was assessed. With enhanced timing resolution, the distortions and artifacts were reduced effectively. The further improved TOF capability also shows a noticeable improvement of detection performance for low uptake lesions, as well as the recovery speed of lesion contrast, which is of practical significance in the lesion detection task.

## Introduction

The clinical positron emission tomography (PET) scanner is generally built with hundreds of detection modules and tens of thousands of electronic channels to provide a transverse field-of-view (FOV) which is roomy enough for most adults’ torsos. Nowadays, the PET scanner’s patient bore often has a diameter of about 70 cm [1]–[3]. It is capable of accommodating a patient weighting up to 200 kg although most of patients are much slimmer. Meanwhile, the region-of-interest (ROI) for imaging is usually a small area inside human body, sometimes a specific organ or an even smaller subregion. It seems wasteful that PET scanner’s FOV must be sufficiently spacious but is rarely made full use of. Furthermore, a large FOV not only results in high manufacture complexity and cost, but also causes performance degradation. The spatial resolution will be affected due to photon alinearity as the ring diameter increases and the sensitivity reduced by the decrease of solid angle coverage. Consequently, it is natural to conceive a PET scanner with an FOV which is comparable in size to the target ROI. Such a design may provide several benefits. Firstly, the FOV will be much smaller but fully utilized, making the system more cost effective. Secondly, upgrading the system for higher performance becomes more affordable, as much less number of detection modules are involved. The last but not least, the system’s size is significantly reduced and make it possible for portable or bedside PET imaging.

Motivated by above observations, we come to the idea of fulfilling such an ROI-focused PET system by two parallel panel detectors. The detectors can be moved according to the ROI’s position, that is a feature of ultrasound or planar X-ray imaging. The panel’s distance can also be adjusted to suit the object’s size, just like in positron emission mammography [4]–[7]. Altogether, the panel configuration allows a PET FOV to be adaptive to the target ROI. Meanwhile, panel PET’s concise structure will greatly reduce the manufacture complexity and make it convenient in applications like real-time PET guided biopsy, intra-operative guidance and monitoring, portable or bedside imaging, and simultaneously multi-modality imaging.

There are two issues to be considered in the image reconstruction of this system design. Firstly, the dual-panel scanner configuration has the inherit limited-view problem, causing severe artifacts in reconstructed images [8]. For this problem, Karp et al have found 200–600 ps time-of-flight (TOF) information effective in reducing artifacts and distortions in reconstructed images of partial-ring PET scanners [9], [10]. However, Karp’s work is dealing with whole-object imaging, while the proposed system is aiming for ROI imaging. Therefore, it is natural and appealing to realize ROI reconstruction here, which has the potential of excluding the influence of high activity structures adjacent to the ROI [11] and accelerating reconstruction speed for real-time imaging applications [12]. ROI reconstruction, as the second issue to be concerned, has been a hot topic in biomedical imaging for a long time. Solid progress has been made in different kinds of imaging modalities, such as the interior reconstruction in computed tomography (CT) [13]–[17], single photon emission computed tomography (SPECT) [18], [19] and omni-tomography [20]. These works have proven that ROI imaging can be achieved via truncated Hilbert transformation (THT) or total variation (TV) minimization, as long as certain additional information is available. Similarly, ROI reconstruction for PET also requires some supplementary data, which is generally the TOF information. Wang has proposed an ROI ordered subset expectation maximization (OSEM) method for TOF-PET using exclusively the line-of-response (LOR) passing the ROI [21]. Kao has utilized the redundancy of TOF-PET data and developed analytic algorithms to perform windowed ROI reconstructions [11]. Although Wang and Kao’s methods are based on full-view data, it is still obvious that TOF capability is crucial in the proposed panel PET design for ROI reconstruction with limited-view problem.

In this work, we investigated the use of dual-head configuration for building an ROI-focused panel-PET scanner. A novel windowed list-mode OSEM algorithm was developed to perform ROI reconstruction, using events originated inside the ROI only. With the proposed scanner design and reconstruction method, we evaluated the impact of TOF on image quality to assess the required system timing resolution (TR).

## Methods

### Scanner Design

**Figure 1:**
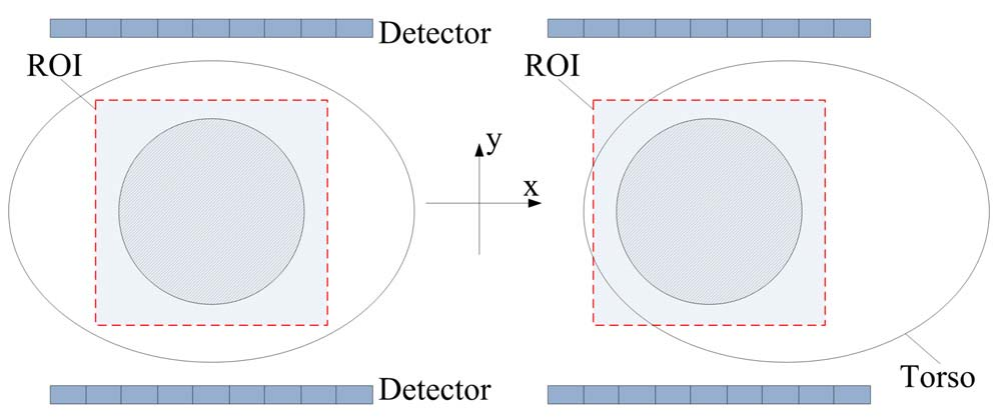
The proposed panel-PET scanner for region-of-interest imaging. The scanner can adjust its position according to different target ROIs.

The proposed PET scanner consists of a pair of panel detectors, one is above the human torso and the other beneath. The two parallel panels can move in both the horizontal and vertical directions to fit the target ROI, while keep being directly opposite to each other. Figure 1 shows the transverse view of the proposed design. For simplicity, we only considered the 2D imaging mode in transverse plane, in which the flight of any gamma photon is restricted. Thus, the scanner’s FOV is defined by the length of and the distance between two panels. The design’s objective is a system so compact that its FOV is barely big enough for imaging the liver – the largest internal organ. For applications with organs or their subregions as imaging objects, such an FOV size will be adequate. Because the liver’s transverse diameter is in the range of 20 to 25 cm, we set the panel detector’s length to 28 cm. Meanwhile, the panel distance is adaptive to patients. It is preferred to deploy the detectors as close to the patient as possible to maximize the solid angle coverage. A large solid angle coverage will not only alleviate the limited-view problem, but also increase the system’s sensitivity. For the case study in this article, the distance between two panels was set as 30 cm according to the average normal adult torso depth. Each panel detector consists of 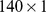 LSO crystal elements in size of 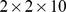 mm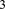. Narrow crystal elements were used to achieve good spatial resolution and then improve the detectability of small lesions.

### Windowed List-mode OSEM

The transverse diameter of an adult’s torso usually ranges from 30 to 60 cm, while the panel detector’s length is 28 cm. The proposed system’s FOV cannot entirely cover the patient’s torso and it never means to. Furthermore, the system is aiming for imaging only the target ROI, which is just a patch of the FOV. Thus, it is natural to use an ROI reconstruction method for the proposed panel PET. Utilizing the TOF information, we developed a windowed list-mode OSEM algorithm, which is capable of ROI reconstruction with panel PET’s inherent limited angular coverage.

The conventional list-mode based EM with TOF information [22] can be formulated as
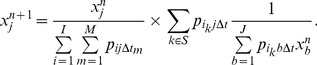
(1)



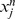
 is the image intensity of pixel 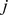 after the 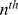 iteration. 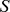 is the complete data set containing all coincidences. For each coincidence, the difference between the two singles’ arrival moments is defined as 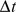. All the measurable time differences are presumed to be discretized into 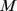 time bins. 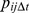 is the probability of an emission from pixel 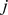 being detected by crystal pair 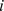 with a certain time difference 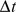. The term 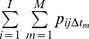 stands for the total probability of an event from pixel 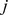 being detected by all possible crystal pairs and with all measurable time differences, which is in fact the pixel’s sensitivity intensity. 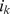 is the index of the crystal pair which detected the 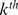 event. Besides, 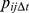 can be factorized into a product of two independent components, which are the 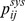 representing the system’s detector and geometric efficiency and the 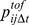 reflecting the TOF probability. The 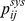 can be obtained by GATE simulation [23], [24]. The TOF probability 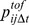 is computed from a timing spread function along the LOR 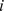, which is modeled as a Gaussian function with full width at half maximum (FWHM) determined by the system timing resolution (TR).

**Figure 2:**
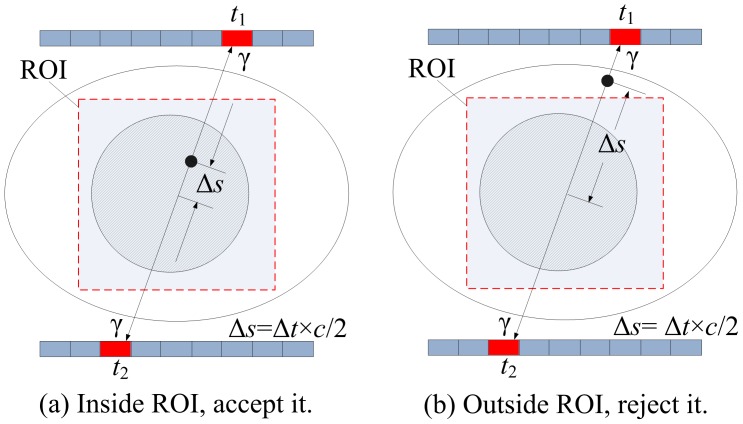
Event discrimination in data acquisition. 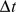
 indicates the arrival time difference of two photons, 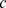 is the speed of light, 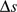 is the distance between the annihilation position and the midpoint of line-of-response.

For ROI imaging, we modified the data acquisition procedure to filter the arriving events. Once a coincidence event is obtained, we calculate its annihilation position along the LOR based on the arrival time difference of the two gamma photons. The event is accepted only if the resulting position is inside the ROI, which is denoted as 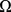. Otherwise, the event is just ignored. The set of all acquired events is defined as 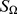. This process is illustrated in Figure 2. Afterwards, the ROI is reconstructed from 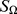 by the ROI version of list-mode EM algorithm, which is expressed as follows:
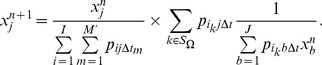
(2)


**Figure 3:**
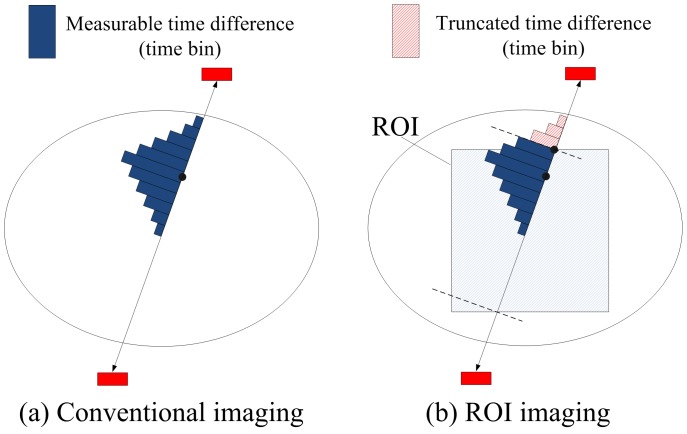
Measurable time differences distribution in conventional (a) and region-of-interest imaging (b). The distribution was assumed as a Gaussian function with full width at half maximum determined by the system timing resolution.

In a conventional TOF-PET system, while a large enough coincidence window is used, all possible time differences are measurable, as shown in Figure 3(a). Thus, there are 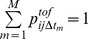 and 
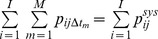
. As to the scenario in ROI imaging as shown in Figure 3(b), the ROI based event filtering truncates the range of measurable time differences. Only time bins associated with locations inside ROI will be measured and included into the sensitivity summation, resulting in a truncated-version of sensitivity intensity 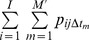.

To speed up the convergence rate, ordered subsets (OS) were used in this work. The events in 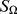 were chronologically divided into 16 subsets and one iteration of the windowed list-mode EM is split into 16 sub-iterations. In each sub-iteration, just one subset of events was selected to update the image. The initial intensity of each pixels in the FOV was set as a uniform positive value. One iteration is accomplished after all the subsets are processed.

### Simulation Setup

**Figure 4:**
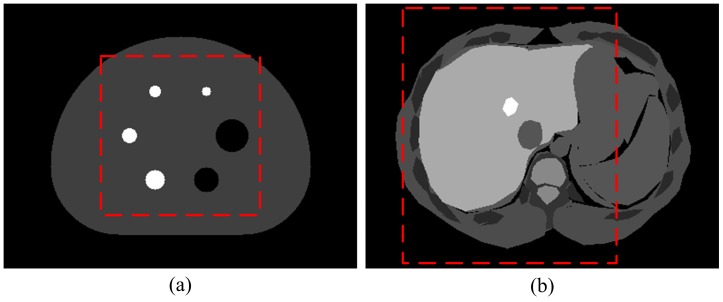
Phantom images with the dotted rectangles as imaging region-of-interest. (a) The NEMA-IQ phantom is about 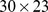 cm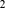 in size, including four hot lesions (10 mm, 13 mm, 17 mm and 22 mm in diameter) and two cold lesions (28 mm and 37 mm in diameter). The region-of-interest size is 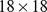 cm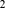. (b) The Zubal abdomen phantom is about 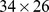 cm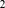 in size. The region-of-interest size is 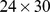 cm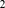.

In simulation study, NEMA NU 2-2001 image quality (NEMA-IQ) phantom [25] and Zubal abdomen phantom [26] were used as imaging objects. NEMA-IQ phantom was employed to evaluate the lesion detectability. As shown in Figure 4(a), it contains four hot and two cold lesions with different diameters, all of which are enclosed in the imaging ROI. The phantom’s background activity is 5.3 kBq/cc and the uptake ratio of hot lesion to background was set as 4∶1. The abdomen phantom in Figure 4(b) is derived from a 2D section of the original Zubal phantom. It was used to simulate a more realistic clinical situation. The image intensities of anatomical regions were arbitrarily assigned. The target ROI contains the entire liver. If the system is capable of imaging the liver with its compact form factor, it should have less difficult in handling other smaller organs. The thicknesses of both phantoms are 2 mm, matching the axial size of the panel detector in simulation.

We employed the Monte Carlo simulation tool GATE (Geant4 application for tomographic emission) package [27] to model the scanner. To reduce the computation cost, we generated list-mode data containing single events with perfect energy resolution and TR at first. These data were blurred afterwards with selected energy resolution and TR to obtain more realistic data [28]. The energy resolution was fixed at 0.13@511 keV, while different TRs were adopted to study the impact of TOF on image quality. An energy window of 400 to 600 keV and a coincidence window of 6 ns was applied to the re-sampled singles for sorting coincidences. In this preliminary study, the main objective is to validate the imaging ability of the system design and hence factors like positron range, photon alinearity, subject attenuation and Raleigh scattering were disabled in the simulation. For image reconstruction, the pixel size was chosen as 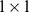 mm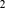, which is half of the 2-mm crystal pitch size.

## Results

### Imaging Feasibility Validation

**Figure 5:**
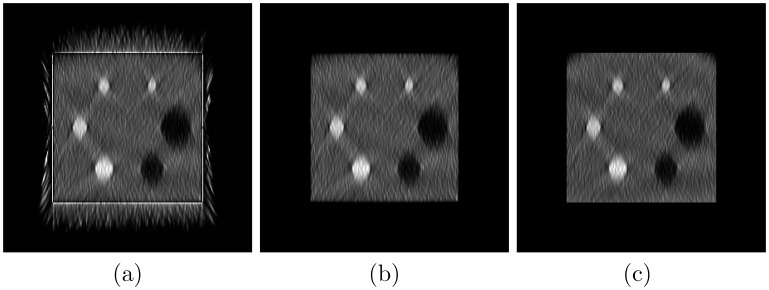
Reconstructed image of the NEMA-IQ phantom obtained by windowed list-mode ordered subset expectation maximization method. (a) The initial reconstructed image of the NEMA-IQ phantom. (b) The reconstructed image after the region-of-interest is cropped out. (c) The reconstructed image obtained by conventional list-mode OSEM method. Note that only the structures inside the ROI are displayed.

The imaging feasibility of the proposed system is demonstrated by the reconstructed NEMA-IQ phantom image in Figure 5, which is obtained by windowed list-mode OSEM with 300-ps FWHM TR. Figure 5(a) is the initial outcome of the reconstruction. The ROI is surrounded by high intensity artifacts, including a rectangle matching the ROI’s edge. Since it is the image inside ROI that matters, only this region is to be displayed. The resulted ROI image is shown in Figure 5(b). All the six lesions can be clearly identified, including the smallest one with diameter of 10 mm. However, all lesions are stretched in various degrees along the direction vertical to the panel detectors, while no obvious distortion is observed in the direction parallel to the detector panel. Besides, X shaped diagonal streak artifacts with rather uniform included angles were observed around each lesion. It seems that both the distortions and artifacts are related to the limited angular coverage.

**Figure 6:**
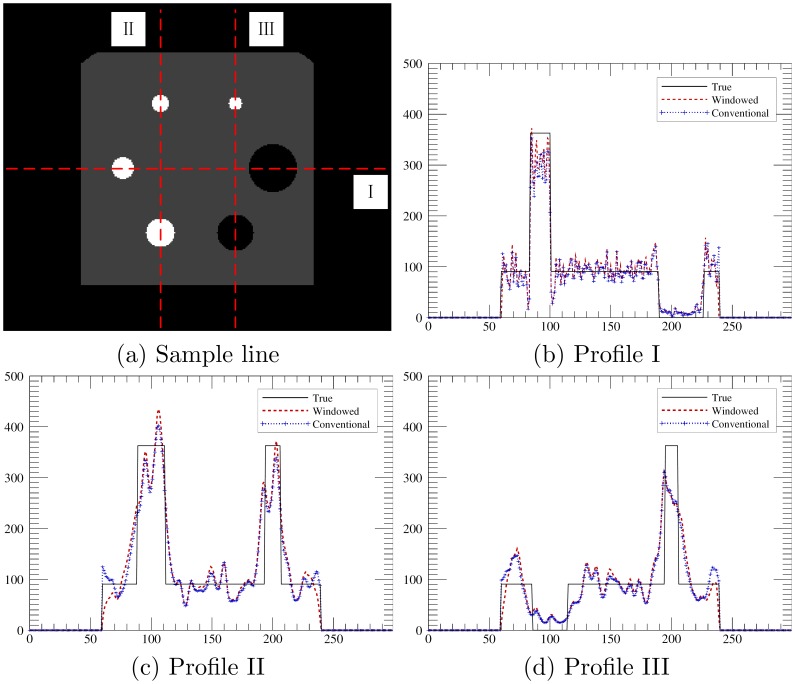
Sampled intensity profiles of the reconstructed image shown in Figure 5. The sample lines in (a) show the locations of the profiles.

The intensity profiles were plotted for further quantitative analysis in Figure 6. The profiles are mainly consistent with the true value. There is no obvious mis-localization of lesions. Meanwhile, the lesion boundary of profile I in Figure 6(b) seems to be more accurate than that of profile II in Figure 6(c) and profile III in Figure 6(d). That agrees with the observations that there are more distortions in the vertical direction than in the horizontal direction. The varying degrees of distortions are also the cause of anisotropic visual representation of structures of interest, such as the shape and size of lesions. Such anisotropism, as well as the pattern of X shaped artifacts, should be put into consideration when the image is further interpreted for lesion characterization.

For comparison, we also included the reconstruction results of the conventional OSEM in Figures 5 and 6. Only trivial differences were observed in the resulted images and profiles. Meanwhile, 4.2 million coincidence counts were used for traditional OSEM and 3 million used for the windowed version. There was a 30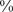 reduction in the data required for the proposed method.

### Impact of TOF on Image Quality

**Figure 7:**
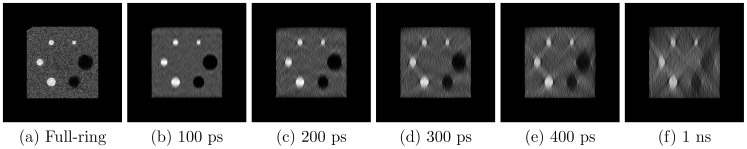
Reconstructed images of NEMA-IQ phantom obtained from TOF-PET data with different timing resolutions. Note that only the structures inside the ROI are displayed.

**Figure 8:**
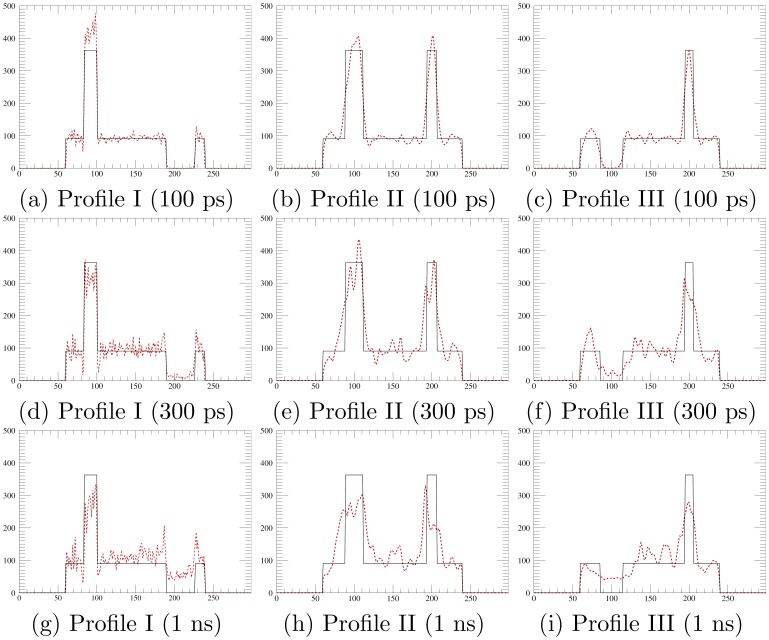
Sampled intensity profiles for NEMA-IQ phantom obtained from TOF-PET data with different TRs. The profiles were obtained from the reconstructed images shown in Figure 7.

To evaluate the impact of TOF on image quality, we reconstructed images for NEMA-IQ phantom from TOF-PET data with different TRs, ranging from 100 ps to 1 ns FWHM. For comparison, a conventional full-ring PET scanner was modeled as well. The Siemens Biograph mCT PET scanner was taken as the prototype while its parameters like crystal size, energy resolution, energy window and etc. were configured to be the same as those of the panel-PET. The results are compared in Figure 7. For image with the 1-ns FWHM TR, all lesions are suffering from obvious artifacts and distortions. There are degradations in image resolution and contrast, as well as loss of structural details. As mentioned above, these problems appear mainly in the direction vertical to the detector panel. Such image quality deteriorations are gradually alleviated by the improvement on TOF capability. With a 100-ps FWHM TR, the artifacts and degradations were almost eliminated, especially the vertical distortions of lesions. Those conclusions were also supported by the numerical evaluation on intensity profiles for different TRs in Figure 8.

**Figure 9:**
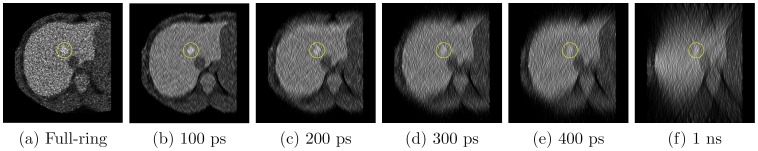
Reconstructed images of Zubal abdomen phantom obtained from TOF-PET data with different timing resolutions. The circle shows the low uptake lesion inside the liver. Note that only the structures inside the ROI are displayed.

The reconstructed images for Zubal phantom with different TRs were compared in Figure 9. Once again, the distortions in the vertical direction resulted in degradation of resolution and loss of structural details. It was observed that liver was stretched along the vertical direction. The counts from high-uptake liver region were mis-located into the surrounding low-uptake muscle region, thus the structure information of liver/muscle boundary was difficult to recognize. With enhanced TR, the visual detectability of the boundary was gradually recovered. Furthermore, the better TR also improved the visual detection performance of the low-uptake lesion, which is inside the liver and has a 1.5 lesion-to-background uptake ratio.

**Figure 10:**
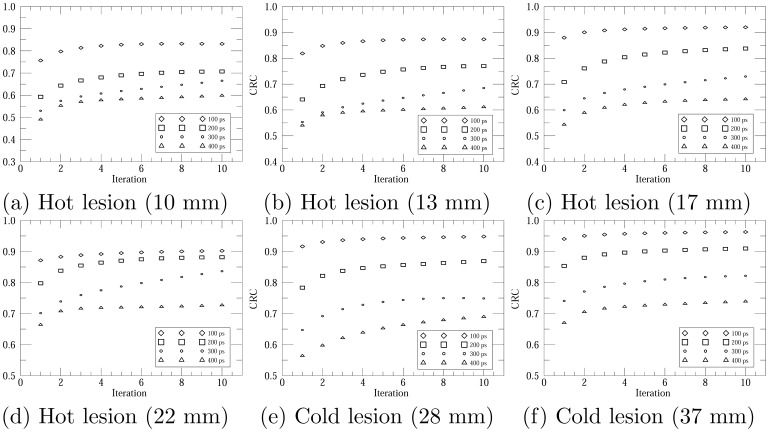
Contrast recovery coefficients versus iteration number for the TOF-PET data with different timing resolutions. To avoid misleading impacts introduced by artifacts and distortions on contrast recovery coefficient values, only image results for 100–400 ps full width at half maximum TOF-PET data were analyzed here.

We also analyzed the contrast recovery coefficients (CRCs) for different lesions to characterize the lesion detectability. Circular region at the center of NEMA-IQ phantom with a diameter of 6 cm was chosen to estimate the background counts. The CRC of hot lesion was calculated as
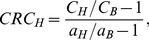
(3)and the cold lesion as



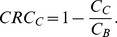
(4)

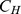
, 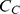 and 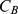 are the average intensities of hot lesion, cold lesion and background regions, respectively. 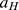 and 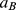 are the true values of the hot lesion and background regions, respectively. Figure 10 shows the CRCs versus iteration number for different lesions. For all lesions, the improved TR leads to a higher CRC value. Furthermore, for most lesions, it seems that a better TR also provides a faster contrast recovery speed. This is more obvious for the results from the TOF data with 100 ps TR, while its corresponding CRC curves usually converge after 2–3 iterations.

Apparently, TOF capability is vital for the imaging feasibility of the proposed ROI-focused panel-PET system. Nowadays, the state-of-the-art TR can reach ∼220 ps for an LSO-based detector module [29] and ∼520 ps for commercial systems [1], [30]. A 300–400 ps TOF capability seems to be achievable in the foreseeable future. Equipped with such a TOF capability, the proposed system can clearly detect lesions as small as 1 cm in diameter, without any mis-localization. Although the artifacts and distortions persisted, their patterns are predictable in certain degree.

## Discussion and Conclusion

In this work, we proposed a panel PET system design for ROI-focused scanning on human torso. The conceptual scanner has a concise geometry and adjustable FOV, which may generate novel applications like portable or bedside PET imaging. To fulfill the ROI imaging, we developed a windowed list-mode OSEM reconstruction algorithm. This algorithm will perform a data discrimination based on the TOF measurements to obtain events whose annihilation positions are inside the ROI. We have used the simulated TOF-PET data to validated the developed algorithm. The ROI-tailored data-discrimination procedure has the potential to reduce the scatter and random events originating from the structures outside the ROI. Furthermore, event counts used in reconstruction will be reduced, depending on the ROI size, activity distribution and local detection efficiency. Since the time of list-mode based reconstruction is almost proportional to the number of events [12], the proposed algorithm would be valuable for certain real-time imaging applications, such as intra-operative guidance and monitoring. The benefits and properties of the windowed list-mode OSEM algorithm are worthy of further investigation.

We also investigated the impact of TOF capability on the resulting image quality of the proposed system. The results indicate that the system with a 300 ps FWHM TR can clearly detect and localize lesions as small as 1 cm in diameter. Meanwhile, the remaining artifacts and distortions have predictable patterns. If the patterns can be used as a prior knowledge, those errors’ influence on the image analysis may not be as severe as expected. Furthermore, a further analysis and modeling of those patterns may enable us to develop post-processing techniques to eliminate such errors in the resulting images.
